# Occasional presence of herpes viruses in synovial fluid and blood from patients with rheumatoid arthritis and axial spondyloarthritis

**DOI:** 10.1007/s10067-015-2974-2

**Published:** 2015-05-17

**Authors:** Rubén Burgos, Graciela Ordoñez, Janitzia Vázquez-Mellado, Benjamín Pineda, Julio Sotelo

**Affiliations:** Rheumatology Department, Hospital General de Mexico, Dr. Balmis 148, 06720 Mexico City, Mexico; Neuroimmunology Unit, National Institute of Neurology and Neurosurgery of Mexico, Insurgentes sur 3877, 14269 Mexico City, Mexico

**Keywords:** Herpes simplex viruses, Varicella zoster virus, Viruses in axial spondyloarthritis, Viruses in rheumatoid arthritis, Viruses in synovial fluid

## Abstract

Viral agents have been suspected as participants of immune-mediated disorders. In the case of rheumatic diseases, the synovial joint cavity represents a secluded area of inflammation which could harbor etiological agents. We analyzed by polymerase chain reaction the possible presence of DNA from various herpes viruses in blood and synovial fluid from patients with either rheumatoid arthritis (*n* = 18), axial spondyloarthritis (*n* = 11), or osteoarthritis (*n* = 8). Relevant findings were as follows: DNA from varicella zoster virus was found in synovial fluid but not in blood mononuclear cells from 33 % of patients with rheumatoid arthritis and in 45 % of patients with axial spondyloarthritis but not in patients with osteoarthritis. Also, DNA from herpes simplex viruses 1 and 2 was found both in the blood and in the synovial fluid from 33 % of patients with rheumatoid arthritis. Our results indicate the occasional presence of DNA from herpes viruses in patients with rheumatoid arthritis or with axial spondyloarthritis. However, these findings might represent a parallel epiphenomenon of viral activation associated either with immunosuppressive therapy or with primary immune disturbances, rather than the etiological participation of herpes viruses in these disorders.

## Introduction

Rheumatoid arthritis (RA) is a chronic inflammatory disease of the synovium and other tissues; its origin is unknown but its immunopathology includes T cell and B cell abnormalities leading to autoimmunity. The cause of RA has been linked to the interaction of genetic susceptibility and environmental factors; infectious agents have long been suspected but no definitive data on the role of viral infections has been demonstrated [1–3]. Epstein-Barr virus (EBV), human herpes virus 6 (HHV6), and human parvovirus B19 have been weakly implicated in RA [4–10]; a recent study found no DNA from herpes simplex viruses 1 and 2 (HSV1-2) or varicella zoster virus (VZV) in peripheral blood cells and urine from RA patients [1, 11]; the possible participation of measles virus has also been suggested [12]. A meta-analysis on the relation between methotrexate and viral infections in patients with RA did not show any significant association with VZV, whereas the association with HS was low [13]. However, the advent of novel immunosuppressive agents for the treatment of RA has also increased the risk of associated viral or bacterial infections. An increased risk of zoster in RA patients has been found [1, 3], particularly in subjects older than 60 years and those under novel immunosuppressive treatment regimens [1], specifically, TNF antagonists like adalimumab, infliximab, and etanercept [3]. Similar risks for other herpes viruses have been described in patients under selective immunosuppressive therapy [13, 14].

A study on the synovial fluid and synovial membrane from patients with several rheumatic diseases using polymerase chain reaction (PCR) analysis was demonstrated in few cases of DNA from different viruses, including cytomegalovirus, parvovirus B1, Epstein-Barr virus, and herpes simplex; however, adenovirus or VZV were not found [15]. Thus far, an association between viral infections and axial spondyloarthritis (ASA) has not been reported.

In multiple sclerosis (MS), another immune-mediated disorder, we have reported an association with VZV, including the finding of VZV in cerebrospinal fluid and in mononuclear cells from MS patients during active relapses of the disease [16]; RA and MS share genetic factors such as the DRB1*04 allele known to participate in both diseases, RA and MS [17, 18].

In this study, we aimed to identify DNA from various herpes viruses (VZV, HSV1 and 2, EBV, and HHV6) in the synovial fluid (SF) and peripheral blood mononuclear cells (PBMC) from patients either with active RA, with ASA, or with osteoarthritis (OA).

## Materials and methods

### Patients

A cross-sectional study was made with 15 adult patients with RA and 11 adult patients with ASA including cases with peripheral joint involvement, as well as in 8 patients with OA requiring synovial fluid drainage of at least one knee for therapeutic purposes. Patients were not included in the study if they had any previous trauma as cause of knee swelling or if they had been subjected to synovial fluid aspiration with or without glucocorticoid intra-articular injection in the last 3 months. In addition, patients having any symptom suggestive of infectious disease within the last week were not included. We also recorded demographic, therapeutic, and clinical data (Table 1). The institutional review board at each center approved the study’s protocol; patients agreed to participate in the study by signing an informed consent form.

**Table 1:** Demographic and clinical features of the population included in the study

	Rheumatoid arthritis *n* = 15	Axial spondyloarthritis *n* = 11	Osteoarthritis *n* = 8
Male	3 (18)	7 (64)	4 (50)
Female	12 (82)	4 (36)	4 (50)
Age, years, mean	43 ± 12	36 ± 9	60 ± 8
Disease duration, years, mean	8 ± 6	12 ± 7	12 ± 10
DAS 28 ESR, mean	4.2 (1.0)	–	–
BASDAI, mean	–	3.7 (2.4)	–
Methotrexate	11 (73)	1 (9)	0
Leflunomide	2 (18)	0	0
Sulfasalazine	7 (47)	6 (55)	0
Hydroxychloroquine	2 (18)	0	0
Prednisone	8 (17)	1 (9)	0

Number in parenthesis represent percentages

### Clinical samples

Four milliliters of peripheral blood were treated with 0.02 ml of ethylenediaminetetraacetic acid (EDTA); PBMC were separated by gradient centrifugation with Ficoll, and DNA was extracted with a kit Qiagen and precipitated with isopropanol. Also, 0.3 ml of SF were treated with 400 μl digestion buffer (500 mM tris, pH 9; 20 mM EDTA, 10 mM NaCl, 1 % sodium dodecyl sulfate, 0.5 mg/ml de proteinase K) for 18 h at 60 °C. Afterwards, proteins were precipitated with 100 μl of protein precipitation solution (Qiagen). DNA was precipitated with isopropanol, dried at room temperature, and dissolved in 100 μl of sterile water. No patient had previous history of herpes zoster.

### Determination of DNA from VZV, EBV, and HHV6

The absolute quantification of viral load was made by real-time PCR. The primers and probes (Table 2) were derived from VZV, strain ORF 31 (gene gB), Epstein-Barr virus (gene gp85), and human herpes virus 6 (gene U29), and designed using Primer Express Software (Ver. 2.0); they were synthesized by Applied Biosystems (Mexico). The TaqMan probes were purchased from Applied Biosystems (Mexico). Each 25 μl of PCR mixture contained 100 ng of DNA either from blood mononuclear cells or from SF in 5 μl of distilled water, plus 12.5 μl of TaqMan universal PCR master mix (Applied Biosystems) and 1.25 μl of primer mixture with a final concentration of each primer of 0.9 μM and of each probe sequence of 0.25 μM. The TaqMan RNAse P was performed to normalize each sample. In addition, reaction mixtures containing the appropriate probe and primer system but without DNA were run on all plates by triplicate and used as negative controls. The PCR mixtures in 96-well microtiter plates were first incubated at 95 °C for 10 min, followed by 50 two-step cycles at 95 °C for 10 s and at 60 °C for 1 min, using an ABI PRISM 7500 real-time PCR system (Applied Biosystems). For each reaction, real-time fluorescence values were measured as a function of the quantity of a reporter dye (6-carboxy-fluorescein [FAM]) released during amplifications. A threshold cycle (Ct) value for each sample was determined as the number of the first cycle at which the measured fluorescence exceeded the threshold limit (10 times the standard deviation of the baseline). Ct values observed for DNA samples were used to calculate the VZV concentration based on the standard curve for the plasmid-containing test sequence.

**Table 2:** Oligonucleotide primers

Viral target	Gene	Forward primer	Reverse primer	TaqMan probe	Product size (pb)
VZV	ORF-31	5′-CACAAAAACACCCGACTCGAA-3′	5′-AAT GGC ACG AAC TCA ACT G-3′	1299 T	65
EBV	gp85	5′-TCCGGCAGGTCCTTCGT-3′	5′-CGAGTGACCGAGAAGGGAGAT-3′	1523 T	67
HHV6	U29	5′-TTGTCTGTTGTCATGCGTCA-3	5′-TCCCATACTGGAGCTTTGCT-3′	713 T	181

### Determination of DNA from HSV1 and 2

The absolute quantification of HSV1-2 viral load was made by real-time PCR using HSV1 and 2 real-time PCR kit (Shanghai ZJ Bio-Tech Co.) according to the manufacturing specifications which included the sequences of the primers used. Briefly, a standard curve was generated by serial dilutions from 1 × 10^7^ copies/ml to 1 × 10^4^ copies/ml. Each 40-μl PCR mixture contained 100 ng of DNA either from blood mononuclear cells or from SF in 4 μl of distilled water, plus 35 μl of reaction mix (Shanghai ZJ Bio-Tech Co.) and 0.4 μl of enzyme mix with 0.1 μl of internal control. In addition, reaction mixtures containing the appropriate reaction mix but without DNA were run on all plates by triplicate and used as a negative control. The PCR mixtures in 96-well microtiter plates were first incubated at 37 °C for 2 min, one cycle; 94 °C for 2 min, one cycle; 93 °C for 15 s and 60 °C for 60 s, 40 cycles, using an ABI PRISM 7500 real-time PCR system (Applied Biosystems). For each reaction, real-time fluorescence values were measured as a function of the quantity of a reporter dye (6-FAM) released during amplifications. A Ct value for each sample was determined as the number of the first cycle at which the measured fluorescence exceeded the threshold limit (10 times the standard deviation of the baseline). Ct values observed for DNA standards were used to calculate the HSV1 and 2 amount based on the standard curve for the positive control included in the kit.

### IgG and IgM antibodies against HSV in SF and in serum

Additionally, after the results of PCR were obtained, the serum and SF were studied for IgG and IgM antibodies to HSV 1. The Enzygnost Anti-HSV1 assays for the detection of HSV-specific IgG and IgM (DiaSorin) were made by the enzyme-linked immunosorbent assay (ELISA) test as follows: Serum samples and SF were diluted 1:100. Samples and controls of 100 μl (negative, positive, and cut-off) were added to each well and incubated for 1 h at 37 °C. After incubation, the plate was washed with wash buffer; 100 μl working enzyme tracer was added to each well and incubated for 1 h at 37 °C. Afterwards, the plate was washed with a wash buffer. Chromogen/substrate of 100 μl was added and incubated for 30 min at room temperature, in the dark. Finally, 200 μl stop solution was added and the plate was read as absorbance values at 450/630 nm using a *Labsystems Uniskan* II plate reader. Samples with absorbance values greater than or equal to the cut-off value were considered positive and negative when the values were less than the cut-off value.

## Results

We studied 15 patients with RA, 11 with ASA, and 8 with OA. Mean disease duration was 8 ± 6 years in patients with RA, 12 ± 7 years in those with ASA, and 12 ± 10 years in those with OA. Demographic and clinical data is shown in Table 1. Most patients with RA were on treatment with disease-modifying antirheumatic drugs (DMARD), methotrexate, sulfasalazine, and prednisone; two on leflunomide; and two on hydroxychloroquine. Six patients with ASA received sulfasalazine, one methotrexate, and one prednisone. Regarding biologic therapy, one patient with RA was receiving rituximab and two with ASA were receiving infliximab.

Two relevant findings emerged from the molecular analysis in search for herpes viruses in PBMC and in SF (Table 3): (1) VZV DNA was found in the SF of a significant number of patients either with RA (33 %) or with ASA (45 %); however, VZV DNA was not found in the PBMC in any case. In contrast, VZV was absent in both PBMC and SF from patients with OA. (2) DNA from HSV1-2 was found in PBMC from 5 (33 %) patients as well as in the SF from 5 (33 %) patients with RA; these viruses were not detected in PBMC or SF from patients either with ASA or with OA (Tables 3 and 4).

**Table 3:** RT/PCR in PBMC and synovial fluid for herpes viruses

Case no.	Diagnosis	PBMC				Synovial fluid			
		VZV (# copies)	HSV1/HSV2 (# copies)	EBV	HHV6	VZV (# copies)	HSV1/HSV2 (# copies)	EBV	HHV6
1	RA	0	0	−	−	0	0	+	−
2	RA	0	0	+	−	772	0	−	−
3	RA	0	0	−	−	1005	0	+	−
4	RA	0	137	+	−	0	0	−	−
5	RA	0	556	+	−	116	80	+	−
6	RA	0	1,400,567	−	−	0	6344	+	−
7	RA	0	710,228	+	−	0	854,725	+	−
8	RA	0	479	+	−	0	641	−	−
9	RA	0	0	+	−	0	0	−	−
10	RA	0	0	−	−	0	0	−	−
11	RA	0	0	+	−	189	0	+	−
12	RA	0	0	−	−	0	0	+	−
13	RA	0	0	−	−	0	0	−	−
14	RA	0	0	−	−	0	0	−	−
15	RA	0	0	−	−	1441	936	−	−
16	ASA	0	0	+	−	2313	0	+	−
17	ASA	0	0	−	−	0	0	−	−
18	ASA	0	0	−	−	1516	0	−	−
19	ASA	0	0	−	−	2252	0	−	−
20	ASA	0	0	−	−	0	0	+	−
21	ASA	0	0	−	−	0	0	−	−
22	ASA	0	0	−	−	0	0	−	−
23	ASA	0	0	−	−	140	0	+	−
24	ASA	0	0	−	−	0	0	−	−
25	ASA	0	0	−	−	0	0	−	−
26	ASA	0	0	+	−	187	0	−	−
27	OA	0	0	−	−	0	0	+	−
28	OA	0	0	+	−	0	0	−	−
29	OA	0	0	+	−	0	0	−	−
30	OA	0	0	−	−	0	0	−	−
31	OA	0	0	−	−	0	0	−	−
32	OA	0	0	−	−	0	0	−	−
33	OA	0	0	−	−	0	0	−	−
34	OA	0	0	−	−	0	0	−	−

**Table 4:** DNA from herpes viruses in PBMC and synovial fluid from patients with rheumatoid arthritis, axial spondyloarthritis, or osteoarthritis

Diagnosis	PBMC				Synovial fluid			
	VZV+ (%)	HSV1/HSV2+ (%)	EBV+ (%)	HHV6+ (%)	VZV+ (%)	HSV1/HSV2+ (%)	EBV+ (%)	HHV6+ (%)
RA (*n* = 15)	0	5 (33)	7 (47)	0	5 (33)	5 (33)	7 (47)	0
ASA (*n* = 11)	0	0	2 (18)	0	5 (45)	0	3 (27)	0
OA (*n* = 8)	0	0	2 (25)	0	0	0	1 (13)	0
Total (*n* = 34)	0	5 (15)	11 (32)	0	10 (29)	5 (15)	11 (32)	0

*RA* rheumatoid arthritis, *ASA* axial spondyloarthritis, *OA* osteoarthritis, *PBMC* peripheral blood mononuclear cells

In the analysis of HSV1-2, a coincidence of positive findings both in PBMC and in SF from RA patients was observed in four out of five positive cases (cases 5, 6, 7, and 8). Also, a high load of viral DNA from HSV1-2 was observed in two patients with RA (cases 6 and 7) as compared with the other positive cases, who had a far lower viral load (cases 4, 5, 8, and 15) (Table 3). Analysis of antibodies (IgG and IgM) against HSV did not show correlative results in serum and SF, nor among positive and negative cases in the PCR analysis; results were positive in about 50 % of all samples, regardless on the group source of the specimen (results not shown).

Positive results for DNA from EBV in both PBMC and SF were frequent in patients from all three groups, although the percentage was higher but statistically non-significant for RA patients (47 % in PBMC and 47 % in SF) than for ASA (18 and 27 %) or OA patients (25 and 13 %). Also, a high concordance was observed between positive samples for EBV in PBMC and in SF (Table 4). The findings of positive cases for EBV in PBMC were similar to those found by us in healthy controls in previous studies [16].

## Discussion

Our study discloses some intriguing features in regard to the potential participation of herpes viruses in RA and ASA. First, the conspicuous participation of a herpes virus in any of the three disorders studied was not observed (RA, ASA, or OA). Nonetheless, herpes simplex virus was found in one third of RA cases; the simultaneous presence of DNA from HSV1-2 in blood and in synovial joint cavity of most positive cases (four out of five) indicates a possible association of HSV with the immunological disturbances of some patients with RA; nevertheless, the relatively low frequency of positive cases does not support the idea of an etiological link. The finding of viral DNA in the SF of the actively inflamed joint might have two explanations, either the viral DNA was passively carried by leucocytes migrating from the blood to the SF or HSV was in actual replication within the synovial membrane as an epiphenomenon related with the systemic immune disturbances associated to RA. Less probable would be a direct etiological link of HSV1-2 infection as a trigger for the immune-related pathophysiology of RA; the most relevant argument against this speculation is the absence of these viruses in two thirds of RA cases studied. Currently, we are searching by ultrastructural studies the possible presence of viral particles in the SF of positive cases for PCR analysis, which would indicate an actual local replication of viruses, rather than the passive carriage of viral DNA by blood leukocytes to the synovial space. It is noteworthy that the amount of HSV copies was rather high in PBMC and SF from two patients with RA (cases 6 and 7, Table 3); however, the absence of these viruses in other similar cases precludes the speculation of a potential etiological participation.

Also difficult to explain is the finding of VZV DNA in the synovial joint cavity in about one third of patients either with RA or with ASA, whereas no viral DNA was found in the PBMC from the same patients. In another immune-mediated disorder, multiple sclerosis (MS), we have demonstrated a high content of VZV DNA and viral particles within the cerebrospinal fluid, which, similarly with the synovial space in RA, the subarachnoid space in MS represents a secluded area related with the pathophysiology of the disease [16]. The finding of VZV in a high percentage of MS cases was restricted to the relapse period of disease. However, in comparison with MS cases, in RA patients, the finding of VZV DNA was infrequent even when all clinical samples from RA patients included in this study were taken during periods of inflammatory activity. Additionally, in the case of RA and ASA, the absence of VZV DNA in two thirds of cases, and the absence of VZV DNA in the PBMC from those cases that were positive in SF, as well as the relatively low number of viral copies found within the synovial space makes remote the possibility of a potential causal association of VZV either with RA or with ASA. Nevertheless, the sporadic demonstration of VZV DNA within the synovial joint cavity might also indicate a possible epiphenomenon of viral replication associated either with the primary immune disturbances of these disorders or with the immune depression produced by therapeutic drugs, which are commonly administered to patients with RA or with ASA. In support of this potential explanation is relevant to point out that the three patients receiving biologic therapy, either rituximab, or infliximab (cases 3 RA, 16 ASA, and 18 ASA; Table 3) were positive for VZV DNA in the synovial fluid.

Although an increased risk for zoster has been reported in RA patients, this feature has been noted mostly in older subjects and in patients under immunosuppressive therapy [1, 3, 19, 20]. However, the inverse relation of the risk for RA or ASA in subjects with VZV infections has not been studied, aside from anecdotal reports of cases of transient and ephemeral arthritis at the time of VZV infection or a case of RA remission simultaneous with a disseminated zoster infection [21, 22].

We conclude that HSV1-2 and VZV are occasionally present in patients with RA; VZV is also occasionally present in patients with ASA. No DNA from herpes viruses were found in patients with OA. Although our results do not support the idea of an etiological link between RA or ASA and herpes viruses, a small group of these patients show the actual presence of DNA from these viruses concordant with the inflammatory activity of RA and ASA.
